# Supplementary material for: Are carbon emissions trading and green financial instruments synergistic? -Comprehensive quantitative research based on content analysis
Source: PLoS One. 2024 Mar 7;19(3):e0298601. doi: 10.1371/journal.pone.0298601 (PMC10919601; doi:10.1371/journal.pone.0298601)
Supplement: S1 Data — (ZIP) [file pone.0298601.s001.zip › Data/地级市Z_F工作报告.zip!/╜¡╦╒/╒≥╜¡╩╨/└┤╫╘╣1⁄2╓┌║┼í╛┬φ┐╦╩2╛▌═°í┐_2007.docx]

各位代表：

　　现在，我代表镇江市人民政府，向大会作工作报告，请予审议，并请市政协各位委员和其他列席人员提出意见。

　　去年工作回顾

　　2006年是“十一五”开局之年。在中共镇江市委领导下，全市人民共同努力，经济社会发展取得令人振奋的新成绩，圆满完成市五届人大四次会议确定的各项目标任务。

　　——地区生产总值突破千亿元大关，预计完成1025.3亿元，增长15.2%，标志着镇江发展迈入了新阶段。

　　——财政总收入突破150亿元，实现151.26亿元，增长27.7%，标志着镇江经济发展质量有了新提高。

　　——服务业增加值增长15.4%，连续两年超过地区生产总值增幅，标志着全市经济结构发生了新变化。

　　——高新技术产业提前一年实现“双倍增”计划，科技进步对工业经济的贡献率居全省前列。

　　——15件为民办实事基本完成。

　　一年来，我们坚持以科学发展观统领经济社会发展全局，主要做了以下几方面工作：

　　（一）加快结构调整，国民经济稳健运行

　　坚持以增量投入带动存量调整，增强发展后劲。完成全社会固定资产投资501.2亿元，增长23.8%。地方财政一般预算收入60.12亿元，增长27.6%。

　　农业稳步发展。落实“一免四补”资金1.6亿元，粮食总产量增长9.8%。加快农业结构调整和丘陵山区综合开发，新增高效农业面积8.8万亩。“三资”开发农业项目680个，总投资30.05亿元。新增市级以上农业龙头企业20家、农民专业合作组织31个。111个农产品获得无公害农产品认证、绿色食品标志和有机农产品认证。

　　工业量质同升。预计规模以上工业增加值完成452亿元，增长21.3%。实现销售收入1583.9亿元、利税130.2亿元、利润70.2亿元，分别增长26.2%、32.8%和45%。完成工业性投入300.5亿元，增长27.3%。在建亿元以上项目83个，新增销售亿元以上企业40家。高新技术产业完成增加值147亿元，占规模以上工业增加值的32.4%，比上年提高2.2个百分点。新增中国驰名商标2个、中国名牌5个、国家免检产品11个。30家企业通过清洁生产省级验收。预计单位生产总值能耗下降4%左右。

　　服务业取得突破。市场建设亮点纷呈，农副产品批发市场一期、红豆广场、月星家居广场、望京天地等重点项目建成开业，沃尔玛购物中心、国际工业品城、第一楼街（步行街）、长江新天地等项目加快建设。“万村千乡”工程推进顺利，建成镇村连锁标准店340家。社会消费品零售总额280.1亿元，增长16%。全年接待境内外游客1416万人次，旅游总收入140.4亿元，分别增长19%和17.3%。世业洲旅游度假区、龙恩木屋度假村等一批新旅游景点建设进展顺利，启动乡村旅游行动计划。加快以大港三期为重点的港口建设，建成3万吨级以上泊位11个。港口集装箱吞吐量23万标箱，增长29.2%。金融机构新增人民币存款124.21亿元，贷款107.53亿元。房地产市场健康有序发展。

　　（二）深化改革开放，发展活力不断增强

　　体制改革不断深化。燃气公司与华润集团、恒顺集团与新加坡第一家食品厂实现战略重组。完成恒顺醋业、索普化工、大亚集团股权分置改革，新增光明沃得、大港股份2家上市公司。调整国有资产经营公司体制，新型监管体系初步形成。稳步推进事业单位转企改制，职工利益得到较好保障。成立南山风景名胜区管委会，理顺南山管理体制。

　　开放型经济难中求进。完成合同注册外资16.58亿美元、实际到位外资7.3亿美元，分别增长57.9%和22.6%。新批总投资1000万美元以上项目94个。制定加快省级开发园区建设发展的若干政策，完成开发区发展总体规划。全市开发园区基础设施投入21.9亿元。完成出口26.63亿美元，增长31%。新批境外投资企业10家。

　　民营经济加快发展。新增私营企业3494家、个体工商户1.59万户；新增注册资本53.22亿元，累计突破300亿元。民营企业完成投资267.5亿元，增长32.6%。民营经济增加值占到全市地区生产总值的53.4%。强化服务，为民营企业争取国家、省专项扶持资金1.45亿元。新增中小企业担保机构5家，注册资本翻了两番，担保基金规模提高4.6倍。全市为民营企业提供贷款432亿元，比上年增加73亿元。各金融机构与1万多家小企业签订银企合作协议。

　　（三）加强城乡统筹，城乡面貌有新提升

　　城市建设和管理稳步推进。完成南徐新城区和北部滨水区总体规划。出台集体土地拆迁补偿办法，开工建设拆迁定建房9.2万平方米。新行政办公区筹建工作扎实推进，启动九华山路、团山路建设。北部滨水区企业和码头搬迁全力推进，引航道水利枢纽工程开工建设，金山湖整治工程全面展开。安排9000多万元用于渔民安置。世业洲内环南路竣工，岛内天然气工程建成。一批城市道路建成通车。镇溧、宁常高速公路镇江段建设进展顺利。按照分级分责的原则，形成防违拆违网络，拆除城区违法建设7.86万平方米。城市综合管理和行政执法水平在全省综合考核中位居前列。

　　新农村建设开局良好。全市财政安排涉农支出2.84亿元，增长27%。安排新农村建设引导资金1000万元，落实粮食直补资金2897万元，充实农民创业担保基金2000万元。“百村示范、百村帮扶”工程深入开展。农村新五件实事工程启动。新、改建农村公路268公里，建制村客运班车通达率99.6%。完成200个建制村农民上网工程，成为全国首批农村信息化综合信息服务试点城市。农民实用技术培训、创业培训和劳动力转移培训超过10万人。农村卫生院改造、综合文化站建设、有线电视村村通工程、环境整治等工作均完成年度目标，农村生产生活条件进一步改善。

　　环境保护和生态建设加强。市财政新增环保投入2610万元。在市区开展高污染燃料禁燃区专项整治行动，淘汰4蒸吨以下燃煤锅炉及生活大灶182台。顺应群众呼声，实施谏壁地区大气环境综合整治，关停违法排污企业4家，对31家排污企业提出整改要求，关闭轧石企业40多家。启动化工行业专项整治。完成焦煤集团等主城区企业的搬迁。关闭11个不合格的饮用水取水口。对黄岗、征润洲等饮用水水源地严格落实34条保护措施，关闭、搬迁保护区内污染企业60余家，全市饮用水质达标率100%。完成大港供水工程，受益群众6万多人。全面实施新一轮“限采”工作，关闭矿山企业31家、采石宕口78个，列入首批计划的34家砖瓦窑基本关闭到位。完成成片造林3万亩，市区新增公共绿地51万平方米。句容市创成国家卫生城市和国家环保模范城市，丹徒区创成全国生态示范区。

　　（四）加大投入力度，社会事业协调发展

　　市财政安排社会事业支出3.92亿元，增长21%。科技三项费用9600万元，比上年增加1900万元。科技进步对工业和农业的贡献率分别为48.8%和54.7%。圆满完成国家重大科技专项“城市水环境质量改善与生态修复技术研究及示范”项目。新增省级以上高新技术企业25家、省级高新技术产品85项。新增市级以上企业技术中心、研发中心28家，博士后流动站2家。申请专利3405件，增长64.8%，增幅居全省第2位，其中发明专利465件，增长124.6%。万人专利申请量居全省第3位。优化中小学布局，镇江中学改造工程全面竣工，镇江国际学校建设积极推进。全市农村免除义务教育阶段学杂费。新建社区卫生服务中心（站）16个，街道（乡镇）社区卫生服务中心规划建成率达到100%。搬迁市传染病医院。出台支持文化发展政策，加快“文化镇江”建设。公布第一批市级非物质文化遗产项目46个。茅以升纪念馆、市少儿图书馆新馆建成开馆。新建农村健身设施场地566个，“万村体育健身工程”建设走在全省前列。在十六届省运会上获金牌数位次前移两位。民族宗教、统计、外事、台侨、人防、地震、史志、新闻、档案等工作取得新成绩，人口计生、妇女儿童、残疾人、慈善等事业实现新进步。

　　（五）坚持以人为本，社会保持和谐稳定

　　高度重视民生。城镇居民人均可支配收入14291元，增长15.3%；农民人均纯收入6626元，增长12%。新增城镇就业4.6万人，转移农村劳动力6.46万人，接收1.2万名大中专毕业生就业。城镇登记失业率3.18%，是全省较低的城市之一。全市在岗职工平均工资比上年增长14.8%。市级筹资8.2亿元，在养老金两次提高标准的基础上，确保退休人员养老金及时足额发放。全市安排就业再就业补助资金4000万元、低保补助金1450万元。全市基本养老保险参保人数比上年净增3.9万人；全面实施“人人享有医疗保障”工程，2万多名救助对象进入医保体系，各项社会医疗保险覆盖人群达226万人。新增住房保障家庭252户。新建农村敬老院9所，改、扩建31所。

　　民主法制建设稳步推进。全面开展“五五”普法教育，扎实推进依法治市、依法行政??义荣辱观，大力弘扬新时期镇江精神，出台并实施《镇江市全民科学素质行动计划纲要实施意见》。深入推进“平安镇江”创建活动，社会治安综合治理考核总分居全省第一，“法治江苏合格县（市）区”建设取得阶段性成效。民兵预备役、国防动员和双拥工作进一步加强。出台突发公共事件应急总体预案和专项预案。安全生产形势平稳，事故起数和死亡人数实现“双下降”，荣获江苏省一等奖。高度重视群众来信来访，建立健全社会矛盾纠纷调处网络，进京、去省上访在全省保持低位。

　　政府自身建设加强。市行政服务中心“两集中、两到位”得到进一步深化和完善，一年来共受理申报事项21.98万件，现场办结率达85%，群众一事一评满意率达99.92%。25个乡镇（街道）建成便民服务中心。办理人大代表建议256件、政协提案394件，办结率和解决率进一步提高。制定和落实《镇江市政府部门行政首长问责暂行办法》，加大行政监察和责任追究力度，深入开展治理商业贿赂行动，进一步提高政府的公信力和执行力。

　　各位代表，过去一年，既是国家宏观调控刚性约束明显增强的一年，也是镇江科学发展成效显著的一年。实践中，我们有四点深刻体会：（一）必须坚持科学发展的工作定位。努力推进科学发展观在镇江的具体化，更加重视经济社会协调发展，加快科技、教育、文化、卫生事业发展步伐；更加重视环境保护和生态建设，严格控制各类高污染、高能耗项目，着力解决群众关注的环保热点问题；更加重视社会公平，加强社会保障体系建设，提高保障水平；更加重视城乡统筹，加大对“三农”的扶持，扎实推进新农村建设。（二）必须坚持创新克难的工作导向。在宏观调控力度加大、各种制约因素增多的情况下，积极创新思路，转变增长方式，不断拓展新的发展空间。采取整合资源、挖掘潜力、节约集约利用土地等措施，破解用地难题；通过加快企业上市、引进战略投资者等方法，拓宽融资渠道；充分利用现有产业基础和镇江的特色优势，加快园区规划建设，加强产业招商，培育符合国家产业政策的项目。（三）必须坚持重点突破的工作思路。立足镇江实际，坚持有所为有所不为，扬长补短，集中力量，重点在自主创新、结构调整、服务业发展、“南山北水”建设上寻求新的突破，创造新的亮点，形成新的城市竞争力。（四）必须坚持以人为本的工作理念。把政府正在做的工作与老百姓最迫切需要解决的事情结合起来，把政府追求的目标与老百姓的期盼统一起来，把政府考核的指标与老百姓的实际感受衔接起来，在激发民智、用好民力，解决民生、造福百姓上下更大的气力，花更多的功夫，努力使镇江人民从改革发展中得到更多的实惠。这四个方面，既是我们对新形势下推进镇江更好更快发展规律的新认识，也是今后工作必须坚持的方针。

　　回顾一年来的实践，我们真切感受到，政府的每一项工作，都离不开全市人民的支持和参与；取得的每一点成绩，都凝聚着全市人民的智慧和力量。在此，我代表市人民政府，向全市人民，向所有关心、支持镇江发展的社会各界人士和海内外朋友，表示衷心的感谢并致以崇高的敬意！

　　在肯定成绩的同时，我们也清醒地看到发展中的问题与不足：经济结构性矛盾依然突出，现代服务业比重不高，工业产品附加值偏低，大项目、好项目不多，有效投入不足，面临的资源、环境压力较大；农民持续增收难度加大，在解决困难群体就业、提高困难家庭生活水平等方面，还有大量工作要做；部分政府工作人员在思想观念、工作作风、服务效能等方面，与基层和人民群众的要求还有差距。在当前形势下，苏南发达地区加速提升，苏中、苏北地区加快赶超，对镇江发展形成了新的竞争压力。对这些问题，需要我们保持强烈的忧患意识、危机意识和责任意识。我们将振奋精神、奋发作为，决不辜负人民群众的期望。

　　今年主要工作

　　2007年是镇江全面建设小康社会的决战之年。按照市委五届二次全会的部署，今年政府工作的指导思想是：以邓小平理论和“三个代表”重要思想为指导，以科学发展观统领经济社会发展全局，坚持更好更快发展，更加注重发展质量和效益，更加注重富民惠民，突出结构调整、自主创新和大项目建设，加快转变增长方式，加强环境保护和资源节约，着力构建和谐社会，向着“全面达小康，建设新镇江”的目标奋进。

　　全市国民经济和社会发展的预期调控目标为：地区生产总值增长13.6%；财政总收入增长16%，地方财政一般预算收入增长16%；全社会固定资产投资增长18%；社会消费品零售总额增长14.5%；城镇居民人均可支配收入增长12%，农民人均纯收入增长10%；城镇登记失业率控制在4%以内；单位生产总值能耗下降4%，二氧化硫排放总量削减4%，化学需氧量排放总量削减2.2%。后三项节能减排指标是约束性的，必须确保实现；对其他各项指标，我们将力争完成得更好。

　　同时，办好15件实事：

　　1、新增城镇就业3万人，转移农村劳动力5万人，培训农村劳动力3万人；

　　2、实施市区社会保险万人续保工程；

　　3、完成西津渡后山、狮子山、磨笄山3处山体滑坡治理；

　　4、整治老住宅小区40万平方米以及谷阳路、吕家湾等低洼积水区，改造市区街巷道路2万平方米，治理危房1万平方米，建设廉租房1.1万平方米；

　　5、扩展区域供水范围至高资、辛丰、上党、丁岗、宝堰等乡镇；

　　6、市区新增60辆环保公交车，优化调整公交线路7－8条；

　　7、实施农民健康工程，新建市紧急医疗救援指挥中心；

　　8、免除城市义务教育阶段学杂费；

　　9、新建全民健身工程2个、全民健身点20个；

　　10、升级改造菜市场，建设镇村连锁标准店500家，新建菜篮子生产基地1000亩；

　　11、新建、改造农村公路180公里，建制村客运班车通达率达100%；

　　12、农村食品和药品安全网建设全覆盖；

　　13、新建市救助管理站；

　　14、基本建成市高技能人才公共实训及鉴定基地；

　　15、修缮伯先公园并对市民免费开放（除动物园外）。

　　围绕上述目标和任务，我们将突出抓好七个方面的工作：

　　（一）关注群众生产生活，加快富民惠民步伐

　　鼓励创业富民。把全民创业作为群众增收致富的根本途径，提高经营性、资产性收入在城乡居民收入中的比重。在搭建服务平台，加强创业培训，强化政策指导，提供快速便捷服务，加强信用担保，建设融资服务体系等方面给予具体的支持和帮助，形成浓厚的创业富民氛围。

　　扩大就业安民。深入开展“充分就业街道、社区”和“农村劳动力充分转移乡镇、村”创建活动，累计使全市80%的社区基本创建为充分就业社区，80%以上农村适龄富余劳动力实现转移就业。继续加强农村劳动力培训、下岗职工再就业培训、大中专毕业生就业培训力度，累计培训8万人次以上。重点抓好零就业家庭、特困人群、大中专毕业生和新增劳动力的就业。确保特困家庭毕业生就业率达到100%。完善与经济发展相适应的工资增长机制，企业在岗职工平均工资增长12%以上。

　　完善保障惠民。加大社会保险扩面征缴力度，力争市区养老保险基金当期收支平衡，确保企业离退休人员养老金按时足额发放，确保城镇社会保险覆盖率达95%以上。重点加强困难群体和农村社会保障制度建设，研究被征地农民参加基本养老保险和医疗保险的引导政策，扩大新型农村社会养老保险制度试点范围。继续推进“人人享有医疗保障”工程，努力使95%以上的城乡贫困人员，90%以上的被征地农民，85%以上的城镇老年居民、少年儿童、农民工，以及所有破产关闭困难企业职工和退休人员参加社会医疗保险。落实和完善城镇廉租房制度，扩大住房公积金制度覆盖面，整顿和规范房地产市场秩序，建成面向中低收入家庭的普通商品住宅30万平方米。发展老龄事业，开展多种形式的老龄服务。进一步完善城乡救助体系建设，安排400万元慈善基金，专门用于助医、助学、助残、助老等活动。推进法律援助工作，维护弱势群体合法权益。继续加强扶贫和茅山老区开发工作。

　　（二）推进结构调整，促进产业优化升级

　　狠抓有效投入。顺应国家产业政策导向，围绕打造长三角先进制造业集聚区的目标，进一步实施“3510”工程，做强做大规模经济，“50强”工业企业销售收入占全市规模工业的45%以上。加大工业有效投入，确保全年完成工业投资375亿元以上，同比增长25%。进一步优化投资结构，鼓励发展高技术含量、高附加值、低污染、低消耗的项目。全力抓好136个投资亿元以上、总额达754亿元的重点项目。积极鼓励企业加快境内外上市步伐。力争全年规模以上工业销售突破2000亿元，亿元以上企业超过280家。

　　突出自主创新。坚持以信息化带动工业化，推动经济增长方式从要素驱动型向创新驱动型转变。切实加强以企业为主体、市场为导向、产学研相结合的技术创新体系建设。以推进企业自主创新“百千万工程”为抓手，新建省、市级工程技术研究中心8家，新认定省、市级高新技术企业50家、高新技术产品100项，高新技术产业增??申请量超3000件，加大知识产权的保护力度和品牌战略的实施力度。新增中国驰名商标2个、中国名牌2个、国家免检产品4个。强化技术改造，提升装备水平，加快传统产业高新化步伐。集中力量做强做优“一园四基地”，推进现代装备制造、新材料、电子信息、软件、生物技术及新医药等创新型产业密集发展。建立省级大学科技园，将经十二路两侧规划建成高科技研发制造产业带。

　　提升服务业发展层次。加快发展生产性服务业，大力培育新兴服务业，全面提升传统服务业，培植一批服务业集聚区。大力发展现代物流业，重点建设港口物流园区、镇南公路物流园区和保税物流园区，培育壮大宝华物流等新型现代物流企业，提高物流配套服务水平。加快大港三期、惠龙码头和扬中兴隆港建设。加快发展旅游业。制定《镇江市旅游发展总体规划》，充分挖掘镇江旅游资源优势，打造特色旅游品牌。大力发展乡村旅游业，力争江苏农林科技示范园、扬中渔乐园等创建为国家级乡村旅游示范点，促进自然资源、农村产业资源、乡土文化资源的整合。完成南山风景名胜区规划修编，建成招隐景区三期工程，确保建成国家4A级景区。建设圌山风景区。开工建设世业洲郁金香主题公园和环岛公路。加快商贸流通和大市场建设，确保第一楼街（步行街）、沃尔玛购物中心和国际工业品城一期建成开业，推进华地购物中心建设，市农副产品批发市场全面竣工，建成2－3个社区商业示范区，提升市场的集聚辐射能力。加快发展信息服务业。推进金融改革和创新，鼓励金融机构加大对经济发展的支持力度。

　　（三）加快发展现代农业，扎实推进新农村建设

　　按照中央农村工作会议要求，把发展现代农业、繁荣农村经济作为推进社会主义新农村建设的中心任务。加大财政投入力度，进一步巩固和完善各项支农惠农政策措施。今年全市财政安排支出3.62亿元，增长27.46%。加大农业科技推广和成果转化的力度，提高科技对现代农业的贡献率。在确保粮食安全、稳定家庭承包制度不变的前提下，放手调整农业结构，鼓励发展高效规模农业。按照“调大品牌农业、调高园艺业、调强养殖业、调特观光农业、调优设施农业”的思路，培育壮大优势产业和特色产品，力争新增亩均年收益2000元以上高效农业12万亩。努力建设适合镇江自然生态条件，集农林牧、种养加于一体的产业体系。进一步提升农业生产和经营的组织化程度，新增龙头企业10家、农业经济合作组织20个。强化农产品质量安全管理。加强农田水利建设。推进丘陵山区农业综合开发，提高农业综合生产能力。

　　加强农村基础设施建设和公共事业发展，努力改善生产生活条件。年内实现农村规划全覆盖，抓好中心村、农村集中居住点的规划编制和试点示范，指导农民居住向城镇和新型社区集中。以促进生产发展为重点，深入开展“百村示范、百村帮扶”活动。继续办好农村新五件实事。疏浚县乡河道168条、整治村庄河塘1667个，完成新修农村公路植树绿化。新增农村改厕5万户、“一池三改”户用沼气1000户。农村中小学全面创成省级合格学校。建成12个规范化、标准化农村卫生服务中心。80%的镇、村完成文化站和文化活动中心建设，加快实施有线电视村村通工程。扎实推进“组保洁、村收集、镇清运、县处理”的卫生长效管理机制，切实改善农村卫生环境。

　　（四）突出园区建设，提高招商引资水平

　　加强载体建设。重点突出园区基础设施建设，今年确保投入20亿元以上，新建标准厂房50万平方米。全面落实加快推进省级开发园区建设发展的政策，推动投资、项目向园区集聚，确保开发园区外资贡献率比去年上升10个百分点。各开发园区要进一步完善发展规划，完成控制性详规、细化分区规划和配套专项规划，明确产业和功能定位，指导项目进区落户，逐步形成产业集聚。改善园区服务，建立项目进区快速“绿色通道”。

　　强化招商引资。重点突出项目建设，尤其是大项目建设。主攻世界500强企业和跨国公司驻华机构，吸纳更多龙头型、基地型、质量高的项目落户。优化开放环境，创新招商方式和手段，提高招商水平，全年实际到位外资增长15%以上。围绕优势行业、特色产业，提高产业招商实效。进一步做好对外资企业的服务工作，促进外资企业增资扩股。挖掘农产品深加工、现代物流、中介服务等领域的引资潜力，提高一、三产业的引资比重。完善招商激励机制和用人机制，引进和培养一批素质强、业务精、富有敬业精神的专业招商人员。

　　加快民营经济发展。新增注册资本48亿元，吸纳民资130亿元。进一步优化环境、落实政策、强化服务，引导和鼓励民营企业加大投入、加快科技创新、提升发展层次。鼓励民营资本参与城市公共设施建设和社会事业发展。

　　（五）推进城市建设，提升城市形象品位

　　打造“南山北水”。加快南徐新城建设步伐，开工建设行政办公楼、规划展示馆，完成文化体育会展中心设计，完成九华山路等8条道路建设，完善新城路网框架。加大北部滨水区开发力度，加快推进引航道水利枢纽主体工程，扩建焦南闸，全面实施金山湖退渔还湖、景区建设及防洪工程。

　　完善城市功能。拓展城市对外交通，高质量完成镇溧、宁常高速公路镇江段工程，推进宁杭高速公路镇江段路面施工。开工建设镇泰过江通道。建成镇江至禄口机场（镇江段）、大港至丹阳、高资至丹徒新区的快速通道。实施学府路东段拓宽等一批城市干道建设，着力解决城市交通瓶颈。优先发展城市公共交通。加大城市给排水设施投入，扩建大港污水处理厂，启动丁卯污水截流工程、谏壁污水处理厂部分管道铺设，实施头摆渡泵站增容。完善天然气供应网，完成城市干道燃气管道配套工程。各辖市也要结合实际，加大城市建设力度，努力提升城市形象。

　　加强城市规划和管理。全面完成镇江城市分区规划、近期建设规划的论证和报批工作，编制完成地下空间利用、综合交通、污水截流管网等专项规划和近期建设用地的控制性规划。有序推进老城区和“城中村”改造。完善城管重心下移体制，提高城管处置能力和长效管理水平。加大环卫设施投入，加强行政执法和环卫队伍建设，逐步实现保洁全覆盖。继续开展违法建设、占道经营、乱贴乱画和占道洗车综合整治，加强建成区绿化全覆盖管理，提高亮化水平，美化城市环境。

　　（六）坚持环保优先，加大环境保护力度

　　深入开展专项治理。继续推进市区燃煤锅炉专项整治和谏壁地区大气环境综合治理，着手开展韦岗地区和312国道镇江段两侧综合整治。认真开展化工行业专项整治，着力提高安全生产、环境保护和技术装备水平。加大水环境综合治理力度，加强征润洲、黄岗、龙门口等饮用水源地保护。通过专项整治，空气环境质量良好以上天数保持在80%以上，地表水符合环境功能区划标准，区域环境质量达到全面小康标准。

　　加快生态市建设。实施《镇江市生态市建设规划纲要》，巩固国家环保模范城市建设成果。扬中市、丹阳市达到国家环境保护模范城市标准，丹阳市建成全国生态示范区。建成一批省级环境优美乡镇和生态村。抓好市区道路绿化景观工程和街头小游园建设。加强植被、山体、水系、湿地等生态敏感区域保护，推进沿路、沿河、沿山地区的绿色屏障和防空林建设。整治采石山体，实施宕口复绿。扎实推进冬春成片造林和农田林网建设，全市森林覆盖率达到18%，建成区绿化覆盖率达到40%。

　　加强资源节约利用。大力发展循环经济，抓好索普、丹化等企业省级试点。深入开展企业清洁生产。严格控制新上高耗能项目，做好能源计量工作，积极研发和推广节能降耗技术，加强重点耗能大户的节能改造。全面落实保护耕地的各项措施，加大土地复垦力度，制订行业用地定额标准和投资强度控制标准，健全土地集约利用和收益分配机制。在全社会深入开展以节水、节地、节能、节材为重点的节约活动，加快建设资源节约型和环境友好型社会。

　　（七）促进社会和谐，协调发展社会事业

　　加强精神文明建设。以提升市民素质和文明程度为重点，扎实推进各类精神文明创建活动。加强社会主义核心价值体系建设。深化社会主义荣辱观教育，大力开展“学礼仪、讲文明、树形象”主题活动。加强廉政文化建设。健全社会信用体系，打造“诚信镇江”。提高未成年人思想道德建设水平，关心下一代健康成长。关心支持驻镇部队、武警和国防后备力量建设，再创全国双拥模范城。

　　大力发展社会事业。市财政安排社会事业支出4.44亿元。各辖市（区）政府也要相应加大对社会事业的财政支出力度。加快科教兴市进程，再创全国科技进步先进市。强化重点领域人才资源开发和人才引进工作，推进人才机制创新。坚持教育优先发展地位，全面实施素质教育。促进义务教育均衡发展，进一步提高非义务教育阶段办学水平。建成镇江国际学校、丹徒实验学校、恒美山庄配套学校。推进区域教育现代化，丹阳、扬中通过省教育现代化县（市、区）评估验收。积极支持在镇高校建设。全市财政安排2000万元，用于发展职业技术教育，新培养高技能人才3000人。增加公共卫生投入，加快建设市疾控中心等一批公共卫生项目。搬迁丹徒区人民医院。完善社区卫生服务功能，逐步实现社区卫生医疗机构药品零差率供应，方便群众就医，减轻群众负担。全面落实支持文化事业和产业发展的政策措施，推进基层文化建设，繁荣文艺创作，发展文化产业，加强历史文化遗产的研究、保护和利用。完成茅山新四军纪念馆扩建工程。市区基本完成社区文化中心建设。广泛开展全民健身活动，提高竞技体育水平，加强体??水平，提高出生人口素质，加强流动人口计划生育管理和服务工作。积极做好妇女儿童工作。

　　推进民主法制和“平安镇江”建设。坚持定期向人大及其常委会报告工作、向人民政协通报情况，自觉接受监督。切实办好人大议案、代表建议和政协提案，认真听取民主党派、工商联、无党派人士和人民团体的意见，使人民的意愿在政府工作中得到充分体现。深化“五五”普法，推进“法治镇江”建设。坚持依法行政，促进公正司法。加强社会治安综合治理，巩固“平安镇江”创建成果，再创“全国社会治安综合治理优秀地市”。建成科技强警示范城市。深化和谐社区建设，完善社区基础设施，提升社区自治和服务功能。强化食品药品监管，保障人民群众的健康安全。严格落实安全生产责任制，杜绝重特大事故发生，提高突发公共事件应急救援能力。发挥“大调解”机制作用，做好信访工作，引导群众依法表达利益诉求。

　　各位代表，今年是本届政府履职的最后一年。政府各组成部门和全体工作人员一定要牢记使命，恪尽职守，圆满完成今年各项目标任务，为下届政府的工作打下良好基础。

　　我们要增强责任意识。人民群众求富思变的迫切愿望，时刻提醒我们不忘肩负的重任；周边地区百舸争流的竞争态势，时刻要求我们保持争先赶超的强烈意识。政府全体工作人员必须以对镇江发展负责、对镇江人民负责、对历史负责的态度，始终保持昂扬奋发的精神状态，带着责任抓发展，带着感情帮民富。带头弘扬“三创”精神，争做“三创”典范，带动全社会的创业热情充分激发，创新智慧充分涌流，创优潜能充分释放。

　　我们要提高行政能力。更好更快发展、全面建设小康、构建和谐社会，对政府工作提出了新的要求、新的考验。必须学习现代经济科技知识，提高驾驭市场经济、发展先进生产力的能力；必须学习社会管理知识，提高协调处理社会事务、做好群众工作的能力；必须学习法律知识，提高依法行政、规范办事的能力。以政府行政能力的不断提高，促进全市经济社会的更好更快发展。政府全体工作人员必须立足本职、加强学习、拓宽视野，切实提高岗位素质和能力。

　　我们要提升服务效能。当前，发展环境的竞争，越来越取决于政府的工作效率和服务水平。政府各部门及全体工作人员必须切实更新观念，自觉完成由管理者向服务者的角色转变，把党的政策、政府的要求和群众的意愿结合起来，不与民争利，多为民办事。深化行政审批制度改革，完善行政服务中心和便民服务网络建设，进一步整合公共服务资源，提高行政效能和服务质量。强化政府督查工作，进一步落实行政问责制。加强政务公开，推行电子政务。大力精简会议，减少无谓应酬，降低行政开支，艰苦奋斗、勤俭节约，少花钱、办好事。

　　我们要改进工作作风。执行力是政府工作的生命力。人民群众不仅看政府说了什么，更注重政府做了什么、做成了什么。每项工作不仅要做到目标明确、思路明确，而且要做到措施扎实、效果明显。要力戒形式主义，力戒表面文章，坚持办实事、求实效，坚持真抓实干、埋头苦干，坚持从严治政、勤政廉政，加大反腐败力度，始终保持为民、务实、清廉的形象。

　　各位代表，总体建成全面小康，是镇江经济社会发展的一次历史性跨越。让我们紧密团结，万众一心，在省委、省政府和中共镇江市委的坚强领导下，全面落实科学发展观，推进更好更快发展，向着“全面达小康、建设新镇江”的宏伟目标阔步前进！
